# On the Front Lines of the COVID-19 Pandemic: Occupational Experiences of the Intimate Partner Violence and Sexual Assault Workforce

**DOI:** 10.1177/0886260520983304

**Published:** 2022-06

**Authors:** Leila Wood, Rachel Voth Schrag, Elizabeth Baumler, Dixie Hairston, Shannon Guillot-Wright, Elizabeth Torres, Jeff R. Temple

**Affiliations:** 1 University of Texas Medical Branch, Galveston, TX, USA; 2 The University of Texas at Arlington, TX, USA

**Keywords:** COVID-19 pandemic, domestic violence, sexual assault, occupational stress, telehealth

## Abstract

In the face of increasing risk for intimate partner violence (IPV) and sexual assault during the COVID-19 pandemic, there is an urgent need to understand the experiences of the workforce providing support to survivors, as well as the evolving service delivery methods, shifting safety planning approaches, and occupational stress of frontline workers. We addressed this gap by conducting an online survey of members of IPV and sexual assault workforce using a broad, web-based recruitment strategy. In total, 352 staff from 24 states participated. We collaborated with practitioner networks and anti-violence coalitions to develop the brief survey, which included questions about work and health, safety planning, and stress. We used chi-square, *t*-test, and ANOVA analysis techniques to analyze differences within position and demographic variables. For qualitative data, we used thematic analysis to analyze responses from four open-ended questions. The sample was majority female-identified (93.7%) and essential workers in dual IPV and sexual assault programs (80.7%). Findings demonstrated that since the pandemic began, IPV and sexual assault staff are experiencing more personal and professional stressors, perceive a decrease in client safety, and lack resources needed to help survivors and themselves. Common problems included a lack of food or supplies at home and work and housing and financial support for survivors. There was a 51% increase in the use of video conference for work, which contributed to workforce strain. Reductions in overall service capacity and a shift to remote service provision have implications for both survivors and staff. These findings suggest a critical need for additional training, infrastructure, and support for the IPV and sexual assault workforce. There is an urgent need to classify IPV and sexual assault staff as first responders and address the occupational stress associated with the COVID-19 pandemic.

The COVID-19 (Coronavirus) pandemic has brought unprecedented challenges for large segments of the workforce, including health care providers, educators, and other essential workers. Less discussed, and arguably as or more strained, are the frontline workers focused on intimate partner violence (IPV) and sexual assault survivors. In addition to pre-COVID risks for occupational stress from low pay, work conditions, burnout, and secondary traumatic stress (STS) (Wood et al., 2019; Dworkin et al., 2016; Kulkarni et al., 2013; Slattery & Goodman, 2009), the IPV and sexual assault workforce now have to navigate the challenges of serving clients safely during “stay-at-home” orders with additional risk to their personal health and safety. Preliminary evidence indicates that rates of IPV (Jaramillo, 2020; Piquero et al., 2020) and child maltreatment (Freidman, 2020) have increased during the COVID-19 pandemic, yet service access and reporting to formal entities may have declined (Boserup et al., 2020; Kaukinen, 2020; World Health Organization, 2020). Hotlines across the nation have seen a surge of use from people stuck at home in unsafe situations, with a lack of formal and informal support (Freidman, 2020; National Domestic Violence Hotline, 2020). Given the novelty and fast pace of COVID-19, there is little information about IPV/sexual assault staff needs and experiences during the pandemic, a critical gap considering their role as public health first responders.

Through housing, economic, and support services, sexual assault and IPV agencies serve principal roles in the health and safety of vulnerable families. To address the challenges of reaching survivors in rural areas and youth (Gray et al., 2015), agencies have recently begun adapting advocacy, counseling, and legal supports to be delivered via chat, text, or video (Brignone & Edleson, 2019; Glasheen & Schochet, 2016; Rempel et al., 2019). Many housing programs have also sought to adopt an advocacy model that is “mobile” and meets clients in their homes or location of their choice. These practice shifts, while gaining momentum, were not widespread before the onset of the COVID-19 pandemic (Brignone & Edleson, 2019; Eden et al., 2015; Nesmith, 2018; Rempel et al., 2019). A critical feature of virtual and in-person work in the IPV and sexual assault field is safety planning, or the collaboration between an advocate (supportive staff member) and survivor to develop a personalized plan to address immediate, specific risks faced by survivors of violence, with the aim of reducing the extent and impact of violence and abuse going forward (Campbell, 2002; Davies, 2009; Davies & Lyon, 2014; Messing et al., 2015). Safety planning, along with attending to housing and other basic needs, are critical components to a disaster response to IPV and sexual assault (First et al., 2017) during an event like the COVID-19 pandemic.

The IPV and sexual assault workforce is noted for their dedication to the mission of their work to end violence (Wood et al., 2017)) and “good soldiering” by enduring difficult occupational experiences to meet the needs of the vulnerable population they serve (Bemiller & Williams, 2011). The workforce is primarily female, racially and ethnically diverse, and comprised of, in some estimates, 50% or more of survivors of interpersonal violence (Wood et al., 2017; Bemiller & Williams, 2011; Dworkin et al., 2016; Kulkarni et al., 2013; Slattery & Goodman, 2009). Workers in this field, like other human service sectors, are at high-risk for occupational stress, including burnout and STS (Voth Schrag et al., in Press; Slattery & Goodman, 2009). Recent research with the IPV and sexual assault workforce found higher workload, younger age, experience with microaggressions, and recent life stress to be predictive of burnout and STS (Voth Schrag et al., in press). Further, burnout is associated with higher turnover intention (Wood et al., 2019) while quality supervision, shared power, and coworker support predicted lower STS (Slattery & Goodman, 2009). The global pandemic has brought unprecedented challenges to meeting the needs of IPV and sexual assault survivors, and left the field with little information on how to equip the frontline staff to meet these needs. The context of the pandemic means that the IPV and sexual assault workforce has to shift both practice model and approach; which, for a workforce already under significant strain, may create additional risks for occupational stress. Thus, information is urgently needed to understand the experiences of those working in IPV and sexual assault services during the COVID-19 pandemic. To address this gap in knowledge, we conducted a national survey of the IPV and sexual assault workforce to learn about occupational stress, service adaptation (e.g., approaches in safety planning), and occupational and personal needs during the COVID-19 pandemic.

## Method

We used a broad recruitment strategy to reach any staff, age 18 or older, working in an IPV or sexual assault-oriented agency, including promoting through social media, email, print flier, web announcement, and word-of-mouth by the study team, local agencies, and state coalitions. Three state coalitions distributed the study announcement on various listservs to staff via email and social media. The study was advertised as an anonymous safety and work study for professionals. In all, 352 staff from 24 states and the District of Columbia participated. The survey was administered via Qualtrics. Participants had the option to sign up for a gift card raffle in a separate survey. Survey questions were voluntary, and the research study was approved by the (blinded) institutional review board.

### Measures

The survey tool was developed by the study team in consultancy with practitioner networks and state anti-violence coalitions. Questions on the survey were anchored by two time periods: before and after the COVID-19 pandemic, with the data of March 13 (the date a state of emergency in the home state of the study was established) provided to mark the beginning of the pandemic. The survey included questions on worker demographics (age, race/ethnicity, gender, age, position at work, type of agency), work, and health (have you been tested for coronavirus? Did you lose your job or have your hours/reduced? Did anyone in your immediate family lose their job or have hours reduced?), client safety and safety planning strategies (Thinking about your clients overall: How has their safety from violence, threats, stalking or abuse changed since the Coronavirus pandemic began?), safety strategies employed to help clients since the onset of COVID-19 (Trying to avoid conflict with the people they live with, encouraging them to stay in another home or residence, suggesting using a hotline, chat, or text service from a social service agency), technology use at work before and after the pandemic, occupational stresses, and needs for working with violence survivors during the pandemic.

### Data Analysis

To assess differences between position and demographic variables, groups were evaluated using chi-square, *t*-test, and ANOVA analysis techniques to examine if there were any significant differences between groups. We selected position and demographic variables for analysis that were significant in previous studies (Wood et al., 2017; Kulkarni et al., 2013). Four open-ended questions were analyzed thematically (Braun & Clarke, 2006), through a combination of inductive (e.g., themes identified organically from the data) and deductive (e.g., pre-established themes based on the categories) theme development. Researchers reviewed the codes and generated a list of themes. One team member coded all 1,080 responses based on those themes, including exemplar quotations, and counts of participant comments reflecting each theme. A second researcher reviewed the themes and counts coded by the first researcher. The team then negotiated to consensus around discrepancies related to themes and meanings.

## Results

As shown in Table 1, survey respondents were majority female, working in a dual domestic violence/sexual assault program in one of 24 states, with the majority (81.5%) from the study team state. The average age of participants was 40.4, with a range from 20 to 85. Of those physically going into the office for work, 76.6% reported having adequate personal protective equipment (PPE).

**Table 1.** Participant Demographics.

	*N*	%
Gender		
Male	18	5.1
Female	328	93.7
Other	4	1.1
Race/ethnicity		
Black/African American	29	8.4
Hispanic or Latino/a	72	20.7
Asian or Asian American	9	2.6
White/Caucasian	214	61.7
Multiracial	16	4.6
Additional race/ethnicity	7	2
Type of agency		
Domestic Violence Program	110	31.3
Sexual Assault Program	13	3.7
Dual Family Violence/Sexual Assault Program	195	55.4
	*N*	%
Campus Program for DV/SA	34	9.7
		
Work position	*N*	%
Advocate/case manager	121	34.5
Counselor/therapist	56	16
Prevention educator	29	8.3
Administrator	39	11.1
Leadership	75	21.4
Other	31	8.8
Work location		
Office	63	17.9
Telecommute from Home	162	46.2
Both Office and Home	117	33.3

### Health and Economic Impact of COVID-19

Nearly 5% of workers had lost their jobs or had a reduction in hours since the pandemic began, and 33.8% had someone in the household lose their job or experience a reduction of hours. The vast majority of the sample (96%) had not been tested for COVID-19 during the time of the survey and only 5% had a family member who had been tested. Out of 254 open-ended responses, 147 participants mentioned problems accessing supplies and food. One participant shared “Certain food items and household supplies have been difficult or impossible to obtain.” Another added “Having issues finding cleaning supplies. Also as a diabetic, I can’t find the alcohol pads I use after testing.” Forty participants noted personal financial strain as a concern, including loss of family income, such as “Household income has decreased due to my partner’s work hours reducing significantly due to the Coronavirus pandemic. So far, I have been able to purchase food and have only struggled to find sanitizing supplies.” Another participant mentioned:

As a part-time employee, I have been doing “gig” work—primarily Instacart. Since the pandemic, I have been weary of being in the grocery store as I still am actively working on site at a DV agency. Due to this, I have not been able to make as much side money as I usually do.

Eighteen survey takers referenced increased anxiety and stress as a health impact, sharing the pandemic has been “Very minimal impact for me personally, but have definitely felt very anxious and struggled in that way.” A total of 35 participants stated they had no problems with food or resources since the pandemic began. “I have been blessed and did not experience any problems during the pandemic.” Notable, but less frequent concerns included lack of childcare, health and health care access, and concerns over being an essential worker:

My issue is with childcare. I have two daycare aged children and both myself and my husband are essential workers. I have had to reduce my work hours with the blessing of my employer due to not having access to childcare. This has financially affected my family.

Finally, one participant shared how the lack of supplies impacted her ability to serve survivors, sharing she had “Difficulty purchasing certain food or supplies for home. I am the Director of a clinic providing sexual assault examinations, however, have had difficulty purchasing supplies needed for PPE, hand sanitizers, cleansers, and long shipping times related to this.”

**Table 2.** Work Technology Changes.

	Pre-pandemic		Post-pandemic			
Technologies Used	*N*	%	*N*	%	P*	
Video conference platform like Zoom, Web-ex, or Go to Meeting	25	7.1	204	58.0	.000	
Texting with clients	73	20.7	126	35.8	.000	
Computer chat with clients	13	3.7	75	21.3	.000	
Emailing with clients	193	54.8	235	66.8	.000	
Phone calls with clients	286	81.3	293	83.2	.286	
Skype or Facetime with client from phone	9	2.6	50	14.2	.000	
Other	16	4.5	28	8.0	.014	
					

*Note*. *p-value from paired *t*-test of significance of pre-pandemic versus post-pandemic.

### Work Changes During COVID-19

The large majority of participants (80.7%) reported being considered essential workers related to stay-at-home orders in their home communities. There was a significant difference observed in essential worker status between those with the job title “prevention educator” and those with other job titles. Of those who reported that their job is considered “essential” in relation to stay-at-home orders, 5% identified their job role as prevention educator, compared to 21% of those reporting their job is not considered essential. As shown in Tables 2 and 3, a major work change was an increase in technology use. Participants reported a 51% increase in use of video conferencing with clients since the pandemic began; 15% increase in texting; 17% increase in computer chat; 12% increase in emailing clients; and 11.6% increase in use of video calling (Skype; Facetime).

We found no significant association between work positions and frequencies of technology use. In 280 open-ended responses about how their jobs had changed since the pandemic began, over 90 indicated participants indicated that they transitioned to providing services via telework/telehealth, leading to adjustment in work approach, as one counselor shared:

I now provide remote therapy services. I have had to learn a completely new platform in order to provide services to clients. I have had to adjust my modalities and dependence on non-verbal communication. A lot of the therapy goals have shifted to crisis intervention related to this pandemic.

Several open-ended responses noted the difficulty of the transition to virtual services for clients, including concerns about safety and technology access.

Clients are not as comfortable using the phone or technology for individual sessions. It is not as easy to gauge how clients are doing without being able to see them if they do not have the technology to use an online platform (or choose not to use it).

Another survey taker shared about the safety challenges brought by the transition to telehealth, sharing “Establishing safety with clients while meeting via Zoom is different since before they would come into my office and there were no fears of being interrupted or overheard by the perpetrator.”

**Table 3.** Changes in Technology Use During COVID-19 Pandemic.

Frequency of Technology Use During COVID-19	% Daily	% Once/A Few Times a Week	% One Time
Video conference platform like Zoom, Web-ex, or Go to Meeting	26.8	59.7	13.4
Texting with clients	35.3	59.7	5.0
Computer chat with clients	27.5	49.2	23.2
Emailing with clients	38.3	51.1	10.6
Phone calls with clients	59.8	36.3	3.8
Skype or Facetime with client from phone	15.2	65.2	19.6
Challenge Level of New Technology	% Very Challenging/Challenging	% A Little Challenging	% Not Challenging
Video conference platform like Zoom, Web-ex, or Go to Meeting	44.4	41.8	13.8
Texting with clients	12.3	27.3	59.5
Computer chat with clients	31.9	29.0	39.1
Emailing with clients	11.7	22.8	65.5
Phone calls with clients	17.5	32.2	50.3
Skype or Facetime with client from phone	37.8	35.6	26.7

As noted by 78 participants, a related change was the transition to working remotely from home. This move created personal complications for staff, for example when one respondent “had to move to work remotely from home. Trying to have a private space to conduct tele-therapy as I live in an apartment with my family.” The transition to remote work met additional resource needs, as one participant shared.

I am working from home, so I had to adjust with technology and get internet connected at my home. We have had to gather a new set of resources to refer our clients to because of the pandemic, and our clients have different types of issues related to the pandemic.

Additionally, the transition to working from home created changes in work and coping approaches.

I am grateful to be able to work from home, but it has significantly changed the way I work. A huge part of my self-care at work was chatting with my coworkers after a hard session or just popping into offices to say hi during breaks. Also, not being able to be physically present with clients is challenging, and many don’t want to or can’t do video so we are left with phone counseling which is very different.

Another major work change referenced by 76 participants was the reduced service availability and access for clients, as the COVID-19 pandemic led to “less resources available and more people in greater need.” The transition away from in-person services decreased the ability of agencies to provided core client services. “I conduct assessments face to face, but now have to do them by phone. My assessments were cut in 1/3.” The decline in clients able to use and engage in services created stress for both staff and clients.

I work at a hotline, so people are already in crisis when they call us, but they’re more escalated right now. We’ve been getting a lot more non-dv/sa/ht related calls from the Spanish speaking and immigrant population who are terrified, broke, and need financial resources, of the 10–15 shelters we used to refer to besides ourselves, there are only two INCLUDING US [emphasis is participant’s] that are open as usual. All others have either closed or are only accepting a certain demographic/locality of people. My job has gone from saying “no, but try this” to “I don’t know, but stay connected.” It’s hard.

Additional workplace changes noted increased responsibilities and longer hours (27 participants), increases in safety and health protocols (23 participants), and changed tasks (20 participants).

### Workplace Stress

As shown in Table 4, the vast majority (94.6%) of participants reported their jobs were at least a little bit stressful before the pandemic began. Over 84% reported at least some increase in stress since the pandemic began, with 23.9% reporting work was a lot more stressful. No significant differences emerged for stress since the COVID-19 pandemic based on agency type (sexual assault only; IPV only and dual IPV/sexual assault programs), perception of client safety, and work location (remote/not remote). There were differences in those reporting increased stress by job type (advocate/case manager, counselor, prevention educator, administrator), although these differences did not reach the level of statistical significance (*p* = .05). Stress increased for those reporting both low and high pre-COVID-19 stress levels (refer to Table 4).

**Table 4.** Changes in Job Stress.

	Low Job StressPre-pandemic (*N* = 176)		Moderate to High StressPre-pandemic (*N* = 175)	
Job Stress Level Post Pandemic	*N*	%	*N*	%
No Change in stress level	31	17.6	23	13.1
Higher stress level	145	82.4	152	86.9
				

In responding to what would best help reduce stress at work during the pandemic, 48 participants noted the need for additional resources from their agency. “Getting the funding and support for technology needed for my staff would help reduce my stress at this point.” Specifically, financial resource needs such as “Increased funding instead of cuts in victim services funding, such as VOCA, VAWA and other crime victim streams of funding. I hate to even ask for PPE’s. We made our own masks but not sure how effective they will be.” In 260 unique responses, main themes included a need for better communication (37 participants), more resources for clients (31 participants), and support for worker mental health/addressing occupational stress (28 participants. One participant summed up communication needs, stating they would like “A clear path forward … clarity from leadership about when we are returning to the office and how that will be done safely. Being able to plan around these timelines and guidelines would be very helpful.” With respect to a need for client resources, a participant shared that they would be helped by “Having more resources to give my clients for Financial Assistance and Free Housing and Free Internet and Computers/Tablets.” The following communicates the need for staff support:

I have worked many hours around the clock and on the weekends. We were short-staffed prior to COVID. I can honestly say I have worked tirelessly to make sure all clients and staff are safe and will continue to do so. I just wish I would be able to take rest time but at this time I am unable to do so. Also the challenge of people tiring of the social distancing and keeping the staff focused on continuing to be safe. I also have people call each day and say it is a hoax and everything should go back to the way it was before. Many challenges.

Other needs included childcare, health protocols, flexible schedules, and job security. Additionally, 32 participants stated that there was nothing needed for stress reduction or they did not know what would help. “I think I’m already engaging in everything I can to reduce stress.” Another worker shared:

I am doing well. I think the transition from working in an assigned office space, moving about at our locations, etc., to working at home, was a bit tough. Now that I have acclimated, participated in weekly “Zoom” meetings, participated in self-care webinars, I am doing ok. The storm has passed, so to speak.

### Client Safety

As shown in Table 5, the majority of participants (73.6%) reported that client safety had decreased at least “a little” since the beginning of the pandemic. Participants endorsed a wide variety of safety strategies, including using a text or crisis line, encouraging contact with the police, suggesting the use of social media to connect with others, and conflict de-escalation as the most frequent strategies selected. Participants who reported worse client safety since the pandemic were more likely to endorse 6 or more safety strategies (77% of participants) than those who reported safety levels as the same or improved (60.8% of participants). No differences emerged in number of safety strategies endorsed between those who indicated client safety was the same or improved versus those who indicated client safety had worsened.

**Table 5.** Client Safety and Safety Strategies Discussed.

Changes in Client Safety Since Pandemic Began	*N*	%
Safety has decreased a little	147	44.5
Safety has decreased a lot	96	29.1
Client safety is the same	64	19.4
Clients are safer	23	7.0
Safety Strategies Discussed	*N*	%
Suggesting using a hotline, chat, or text service from a social service agency	279	89.7
Encouraging calling the police	251	82.8
Suggesting using social media and phones to connect with other people	238	79.6
Trying to avoid conflict with people they live with	240	79.2
Offering emergency shelter	236	77.1
Encouraging them to stay in another home or residence	226	76.1
Teaching conflict de-escalation techniques	216	73.0
Encouraging them to stay in another room from people they live with	198	66.4
Encouraging calling CPS	169	59.3
Offering housing programs	157	55.5
Encouraging them to stay off social media (Facebook, Snapchat, Twitter, Instagram)	139	47.9

In indicating what would most help clients, 270 unique responses revealed that financial resources and assistance for clients would be most effective (70 participants), including, “Ability to help them pay their rent/utility, etc.” and “adequate food and money to pay rent.” One survey taker added:

My clients are concerned about their financial status. Some of them have children and are single mothers and they really need financial resources since their jobs have decreased due to the pandemic. Some of them are afraid to go to work because they don’t want to be infected or infect their children. Some others are concerned about their children’s mental health.

Another major theme, referenced by 57 participants, was housing and shelter, including “Housing assistance for when they exit after pandemic” and “increased options for shelter (children’s shelter and family violence shelter).” A participant shared “A lot of housing programs are not even taking applications right now which means residents are staying in shelter longer.”

Long term housing options that are sustainable and funded with limited restrictions. we are seeing survivors with little to no income due to abuse or loss because of the coronavirus, and no ability to get jobs right now which is leading to extended stays in shelter with no transitional options.

Forty-six participants mentioned specific resources, such as transportation and food. Access to increased mental health and technology services was referenced by 24 and 21 participants, respectively. One participant, noting the connection between the two shared they would like “Having more staff to help facilitate group and answer crisis calls. Having a Tech person to assist with computer/internet problems.”

## Discussion

In the face of the coronavirus pandemic, IPV and sexual assault service providers are striving to provide high quality, life-sustaining, and transformative services in a new and challenging context. As advocates learn from and with survivors, there is mounting evidence that COVID-19 has created new pathways for abusive partners to increase their power and control within violent relationships, as well as changes to survivors’ access to formal and informal supports (Fielding, 2020; Kaukinen, 2020). Helping survivors in the midst of a pandemic has made an already challenging job even more taxing—further complicated by the fact that much of this workforce are also survivors. Thus, in surveying staff, we sought to understand changes in service delivery due to COVID-19, experiences of occupational stress by IPV and sexual assault workers, and strategies for safety planning as social distancing and stay-at-home orders continue. Findings indicate that, since the onset of COVID-19, IPV and sexual assault staff have more personal and professional stressors, are challenged by practice adaptations, perceive that their client safety has decreased, and lack the needed resources to help survivors.

### Service Adaptations

Two major service adaptations in the face of the COVID-19 pandemic are clear from both the qualitative and quantitative findings: a reduction in overall service capacity and a move towards remote service provision. Each of these shifts has significant implications for survivors and staff alike. A frequent theme echoed by staff members relates to the combined forces of reduced resources (including fewer available shelter beds due to the implementation of social distancing procedures and reduced financial resources due to the economic downturn) with increased demand for services. Collectively, these factors reduce the capacity of the service sector to meet survivor needs and increase their level of stress; especially alarming given their need to make difficult decisions regarding the allocation of tenuous resources. Increasing financial resources through the use of CARES Act funding and creatively deploying resources made available through state emergency declarations (e.g., using FEMA trailers to increase socially distanced emergency housing capacity) could help agencies address some of these challenges during future pandemics and other crises (e.g., natural disasters). Of particular note is the need for housing for IPV and sexual assault survivors, which was a pressing need prior to the pandemic (Klein et al., 2019). Use of flexible funds and policy protections from evictions may help survivors obtain and keep affordable and safe housing.

Along with reduction in capacity, many advocates reported a shift in the modality of service provision from in person/on-site services to remote, telework, and work from home formats. Nearly half of participants were solely teleworking after the onset of COVID-19, while another 33% were combining telework with some site-based work. Accordingly, there was a substantial increase in the use of new technologies to reach clients, with a marked rise in the use of video conferencing platforms in particular. While staff may have previously used texting or e-mail with clients, over half adopted video conference platforms as a service delivery mode in the wake of COVID-19 for the first time. Video-based technologies are viewed as uniquely challenging by staff, with many participants reflecting on their own anxiety related to fears for clients’ safety and comfort engaging in safety planning or therapy in a digital space. The extent to which these perceived concerns reflect actual safety risks is unclear and deserving of additional research, especially since advocates reported fewer challenges implementing things such as phone, e-mail, and text-based advocacy. In addition to concerns about the risk of the violent partner’s surveillance of sessions and the survivors’ comfort engaging in technology, advocates noted the challenges of conducting this emotionally challenging and necessarily private work from their home. The need for separation between a staff member’s personal and family space and their stressful work environment was echoed by several participants, which could shape how agencies support teleworking arrangements moving forward to prevent STS and burnout.

### Safety Planning With Survivors

Staff was overwhelmingly concerned about the decrease in survivor safety since the onset of the pandemic. While the limited data have been inconsistent (Fielding, 2020; Piquero et al., 2020,), staff indicate that concerns about isolation and increased lethality from stay-at-home orders (Kaukinen, 2020) coupled with reduced resources contribute to more dangerous conditions. The most frequent safety strategies endorsed emphasized connections with formal supports, such as social service agencies and police, followed by de-escalation and risk-reduction strategies. Of particular note is that 83% of participants in the current study suggested reaching out to the police, while a similar study revealed that only 18% of survivors reported contacting the police for a safety concern (Wood et al. in press). This discrepancy reiterates the notion that survivors, especially from Black and Brown communities, may not view police or other formal first responders as safe or supportive avenues to address potentially violent situations. These results also highlight the inherently individually focused nature of safety planning, with no one-size-fits-all set of solutions, but rather a unique mix of strategies needed for each situation. Mixed reports emerged for social media, with staff encouraging its’ use as a tactic for building informal support while also discouraging its use as a potential vector for surveillance or control. Historically, safety planning has been geared toward helping people leave abusive relationships, and focuses heavily on physical escape strategies through formal supports like law enforcement or Child Protective Services. A paradigm shift in the last decade has introduced new mechanisms of safety planning that focus on the needs of those who cannot or do not want to terminate their relationship, or who may not want to engage with law enforcement or other retributive systems (Davies, 2019). Considering the mistreatment of survivors by some law enforcement and other systems, and the context of social distancing and stay-at-home orders, staff may better serve the client by building on the strengths of informal networks and other social service providers.

### Worker Stress

Occupational stress is already a major risk for workers in the IPV and sexual assault service field (Authors, 2019; Dworkin et al., 2016; Kulkarni et al., 2013; Slattery & Goodman, 2009). Like essential workers and first responders from other health and social service fields, COVID-19 has only increased these risks for burnout and STS. Indeed, 85% of respondents in this study reported an increase in workplace stress related to the COVID-19. Burnout and STS are detrimental not only to worker wellness, but contribute to turnover and decreased positive client outcomes (Barak et al., 2001). For many staff members, the drive to do IPV and sexual assault work, despite low pay and hazardous conditions, is to help people get safer and contribute to social justice through helping to end violence (Wood, 2014; Bemiller & Williams, 2011). Doing this important work in person is inherently risky in the context of COVID-19, putting both the survivor and the staff member at risk of the virus. For a mission-driven workforce, who give up prestige, income, and more comfortable working conditions to help build the safety and well-being of violence survivors, the feeling of being a potential vector of risk creates a unique cognitive dissonance for staff. Persistent lack of resources, both in terms of being able to provide direct aid to support survivors and with respect to PPE and technology, create additional stress for staff. Although there was no strong evidence for significant loss of hours or jobs in this sample (which was collected early in the COVID-19 crisis, and may not reflect the reality for staff as the economic impacts for agencies cascade over time), it is important to recognize that over one-third of participants had a family member experience reduced hours or lost jobs. This familial economic strain, compounded by the stress of work in an already comparatively low-paying social service field, could contribute to exacerbating the impacts of occupational stress on IPV and sexual assault workers.

Occupational stressors such as burnout and STS may be exacerbated for first responders during the phases of disaster—mitigation, preparedness, response, and recovery (Fogel, 2017), especially for those working with populations who have histories of trauma and violence. As a global disaster, COVID-19 impacts IPV and sexual assault services providers around the world, creating additional strain and stress in the response to, what for many clients, is a dual crisis of violence and a pandemic. Natural disasters may not only exacerbate risk and intensify severity of violence, but may also delay healing and recovery (First et al., 2017). Previous research has documented the disconnect between emergency responses and vulnerable populations during natural disasters (First et al., 2017; Fogel, 2017). Frameworks for addressing IPV and natural disasters include development of collaborations, increasing awareness, ensuring basic needs are met, provide comfort, and connect to long-term resources (First et al., 2017). These frameworks help to build program capacity to address client needs, but notably leave out strategies to reduce occupational stress risk for workers in IPV and sexual assault settings and are limited to emergency stabilization, rather than ongoing crisis. Given these omissions, results from this study highlight recommendations to improve the response to disasters like COVID-19 in the IPV and sexual assault workforce.

### Recommendations

There is a critical need for additional training, infrastructure, and support for virtual modalities to the IPV and sexual assault workforce. As states struggle with providing services to survivors in high-need urban areas and isolated rural areas, implementing and evaluating safe and effective virtual services will enhance the reach of IPV and sexual assault services beyond the pandemic. Evaluation of virtual services is needed to improve the service delivery medium and provide guidance for the field. There is also an urgent need to not only classify IPV and sexual assault essential workers as first responders, but to address occupational stressors experienced during the COVID-19 pandemic. Given the dearth of guidance on occupational stress intervention strategies for this population, future research should focus on individual and agency level interventions to address burnout and STS. Organizational leaders can learn from the varied ideas presented by study participants related to addressing worker stress during COVID-19. For example, ensuring ongoing, consistent, and transparent communication was a clear recommendation, as was ensuring that staff had access to the resources necessary to carry out their roles. Further, hazard pay, counseling, and assistance with material and resource support such as childcare are essential to minimizing stress impacts in this workforce.

### Limitations

The findings and recommendations stemming from this study should be evaluated in light of a number of limitations. First, the convenience sample of IPV and sexual assault service workers should not be viewed as representative of all IPV and sexual assault staff. The substantial majority come from one Southwestern state, and the sample may over-represent those with easy access to technology or those who have experienced less impact from the COVID-19 and social distancing, and thus had more time or emotional bandwidth to participate in a study. The data collection window captured a snapshot at the beginning of the COVID-19 experience, which can tell us a great deal about that moment in time. However, as practices shift and some changes become entrenched, there is ongoing need for longitudinal research to understand how these impacts are unfolding over time. The survey tool was designed to be brief, which was necessary to reduce participant burden in the middle of a global pandemic. Longer and more established measures would have been preferable to capture additional factors that might have contributed to survivor and staff outcomes. Qualitative follow-up interviews are needed to address many of these limitations, and future work should build on these findings to enhance our understanding of staff experiences and safety planning in the post-COVID-19 service landscape.

## Conclusions

The COVID-19 pandemic presents a challenge for professionals providing services to survivors of IPV and sexual assault. Based on the experiences of the 352 individuals that participated in this study, data suggest that frontline workers, already at risk for high level of occupational stress, are facing even more challenging conditions in providing services to their clients, sometimes risking their personal health and safety, during the COVID-19 pandemic. Since the onset of the pandemic, service providers across the country have seen increased rates and intensity of IPV, sexual assault, and child maltreatment; however, survivors’ ability to access services has declined. Future research is needed to further understand service adaptations, modifications to safety planning with survivors during stay-at-home and social distancing orders, the effect of these conditions on worker stress, and how these will shift in a post-pandemic environment.
